# "Is Cybermedicine Killing You?" - A Response From the Authors of the Cochrane Review

**DOI:** 10.2196/jmir.7.4.e40

**Published:** 2005-07-28

**Authors:** Elizabeth Murray, Jo Burns, Sharon See Tai, Irwin Nazareth

We wish to respond to the paper by Rada [1] and accompanying editorial by Eysenbach and Kummervold [2] in JMIR regarding our Cochrane systematic review on interactive health communication applications (IHCAs) for chronic disease. This systematic review was published in October 2004 in Issue 4 of The Cochrane Library, and we were notified of potential errors in the direction of change for clinical outcomes about 10 days after publication. We immediately reviewed our work and confirmed that errors had been made. We decided that our main responsibility was to limit the harm caused by these errors, by firstly, ensuring that all relevant stakeholders were informed as quickly as possible, and secondly, working toward correcting the errors in a transparent open fashion. Within 48 hours of the first notification, we had informed (a) the editors of the Cochrane Consumers and Communication Review Group, (b) the funders of the work, and (c) the University College London (UCL) press office. For technical reasons, it was not possible to immediately withdraw the review from The Cochrane Library website, so an alert was posted on the review, warning readers that there were errors in the direction of change for some of the behavioral and clinical outcomes and apologizing for the mistake. No one could access the review without seeing this alert. The review was withdrawn in Issue 1 of The Cochrane Library in 2005. We also made every effort to contact journalists who we knew to be writing articles about the original publication, but which had not yet been published.  Since then we have continued to work closely with the editorial team of the Cochrane Consumers and Communication Review Group to revise the review. We are grateful for all the help and support we have received in this process.

We hope to be republishing the revised review shortly. The revision will address issues of the review's scope and the pooling of data that were raised by peer reviewers and also by Eysenbach and Kummervold. We have been greatly assisted by comments from external statistical reviewers from the Cochrane Group and from an internal independent statistical advisor, who have reviewed our methodology. We anticipate and welcome a lively and ongoing debate on these methodological issues.

In the meantime, we would like to correct three factual inaccuracies in Rada's paper. While Rada states that "the coauthors Nazareth and Tai [are] credited with doing the coding," we should clarify that Nazareth was not responsible for coding the data and is not credited with this in the review.  Secondly, Rada writes that the "UCL...news bulletin...remained [on the website]." We should point out that the UCL press release, while still available on the UCL website, is clearly linked to the retraction and subsequent press release summarizing the errors and steps taken to rectify the errors. Thirdly, the UCL press release did define interactive health communication applications, although Rada said it did not.

We agree that the press coverage of the original report was not matched by coverage of the retraction, and we concur that this was unfortunate. However, the Cochrane Collaboration made two press releases advising all media outlets of the errors in October and December 2004.
